# Comparison of registered and published intervention fidelity assessment in cluster randomised trials of public health interventions in low- and middle-income countries: systematic review protocol

**DOI:** 10.1186/s13643-016-0351-0

**Published:** 2016-10-19

**Authors:** Myriam Cielo Pérez, Nanor Minoyan, Valéry Ridde, Marie-Pierre Sylvestre, Mira Johri

**Affiliations:** 1 Centre de Recherche du Centre Hospitalier de l’Université de Montréal (CRCHUM), Montréal, Québec Canada; 2 Département de Médicine Sociale et Préventive, École de Santé Publique (ESPUM), Université de Montréal, Montréal, Québec Canada; 3 Institut de Recherche en Santé Publique Université de Montrèal (IRSPUM), Pavillon 7101 Avenue du Parc, Centre-ville Station, P.O. Box 6128, Montreal, Quebec H3C 3J7 Canada; 4 Département de Gestion, d’évaluation, et de Politique de Santé, École de Santé Publique, Université de Montréal, Montréal, Québec Canada

**Keywords:** Cluster randomised trials, Implementation fidelity, Public health interventions, Process evaluation, Developing countries, Systematic review protocol

## Abstract

**Background:**

Cluster randomised trials (CRTs) are a key instrument to evaluate public health interventions, particularly in low- and middle-income countries (LMICs). Fidelity assessment examines study processes to gauge whether an intervention was delivered as initially planned. Evaluation of implementation fidelity (IF) is required to establish whether the measured effects of a trial are due to the intervention itself and may be particularly important for CRTs of complex interventions. Current CRT reporting guidelines offer no guidance on IF assessment. We will systematically review the scientific literature to study current practices concerning the assessment of IF in CRTs of public health interventions in LMICs.

**Methods:**

We will include CRTs of public health interventions in LMICs that planned or assessed IF in either the trial protocol or the main trial report (or an associated document). Search strategies use Medical Subject Headings (MESH) and text words related to CRTs, developing countries, and public health interventions. The electronic database search was developed first for MEDLINE and adapted for the following databases: EMBASE, CINAHL, PubMed, and EMB Reviews, to identify CRT reports in English, Spanish, or French published on or after January 1, 2012. To ensure availability of a study protocol, we will include CRTs reporting a registration number in the abstract. For each included study, we will compare planned versus reported assessment of IF, and consider the dimensions of IF studied, and data collection methods used to evaluate each dimension. Data will be synthesised using quantitative and narrative techniques. Risk of bias for individual studies will be assessed using the Cochrane Collaboration Risk of Bias Tool criteria and additional criteria related to CRT methods. We will investigate possible sources of heterogeneity by performing subgroup analysis. This review was not eligible for inclusion in the PROSPERO registry.

**Discussion:**

Fidelity assessment may be a key tool for making studies more reliable, internally valid, and externally generalizable. This review will provide a portrait of current practices related to the assessment of intervention fidelity in CRTs and offer suggestions for improvement. Results will be relevant to researchers, those who finance health interventions, and for decision-makers who seek the best evidence on public health interventions.

**Electronic supplementary material:**

The online version of this article (doi:10.1186/s13643-016-0351-0) contains supplementary material, which is available to authorized users.

## Introduction

As evidenced by their growing presence in the scientific literature [1, 2], cluster randomised trials (CRTs) have become a key instrument to evaluate public health interventions [1, 3–7], particularly in low- and middle-income countries (LMICs) [3, 8]. Randomised controlled trials (RCTs) are widely considered to provide the highest quality of evidence on the effectiveness of health interventions [9–12], and CRTs are a form of randomised trial in which clusters of individuals (such as families, villages, hospital services, or schools) rather than independent individuals are randomly allocated to intervention or control groups [2]. Increasingly, public health researchers recognize the importance of developing health interventions that are directed not only to individuals but also to populations, communities, and a wide range of social and environmental factors influencing health [13, 14]. CRTs offer an appropriate design to assess such public health interventions and also to measure the overall effect of an intervention at the population level [3, 5, 8, 13, 15], heterogeneity of impact among population subgroups, and equity [16, 17].

### Implementation fidelity in CRTs of public health interventions

Although the scientific debate is ongoing [18], randomised trials are generally viewed as the gold standard for establishing evidence of intervention effectiveness. Despite this, the use of CRTs to evaluate public health interventions raises unique methodological challenges. Recent systematic reviews of CRT methods have found evidence of improvements in the design and analysis of CRTs while noting deficiencies in trial implementation that may compromise their validity [19, 20]. Previous systematic reviews have emphasised the importance of process evaluation to mitigate these methodological problems, which can affect the internal and external validity of trial results [3, 19, 21–23].

“Implementation fidelity” refers to the degree to which an intervention is delivered as initially planned [24]. Fidelity assessment is an aspect of process evaluation that aims to understand and measure to what extent the intervention is being implemented as intended, with a view to clarifying relationships between intervention and its intended outcomes, and learning what specific reasons have caused the success or failure of the intervention [9, 24, 25]. Evaluation of implementation fidelity within trials has multiple benefits, which may include increased confidence in scientific findings, increased power to control for confounding factors and detect intervention effects, and increased ability to evaluate the performance of an intervention based on theory [26]. Several studies have found that interventions implemented with high fidelity achieved better results in comparison with low-fidelity interventions [27–33]. Fidelity assessment can improve the internal and external validity of CRTs [19] by providing evidence that the trial results are due to the intervention itself rather than to confounding variables and facilitating generalization of results to contexts that may differ substantially from the original trial setting [9, 24]. Fidelity assessment may be particularly important for trials of public health interventions, as these interventions tend to be complex and constituted by multiple components [10, 34] that may act independently or interdependently [35], leading to a greater potential for variation during implementation [24].

### Framework for the evaluation of implementation fidelity used in this review

Table 1 outlines the conceptual framework for evaluation of implementation fidelity used in this review. The framework is based principally on the work by Carroll et al. [24] and includes elements of implementation fidelity and moderating factors that may affect the delivery process. The framework was further refined by Hasson, who expanded the list of moderating factors considered in the framework [36]. We selected this framework to guide the review because it provides a comprehensive synthesis of previous work on implementation fidelity and has been widely influential.

**Table 1:** Conceptual framework for implementation fidelity used in this review

Fidelity components	
Content	Defined as an attempt to establish the “active ingredients” of the intervention, for example, in a theory of change or logic model, and assess whether they have been delivered as planned
Coverage	Refers to the degree to which all persons who met study inclusion criteria received the intervention
Frequency	Refers to whether the intervention was delivered with the regularity or frequency planned by its designers.
Duration	Establishes whether the intervention was delivered with the duration planned by its designers.
Moderating factors	
Comprehensiveness of intervention description	Factors such as the degree of intervention complexity and whether the intervention description is complete or incomplete, vague, or clear may influence the degree of implementation fidelity.
Strategies to facilitate implementation	Several support strategies may be used to optimise and to standardise implementation fidelity.
Quality of delivery	Concerns whether an intervention is delivered in a way that increases the likelihood of achieving the desired health outcomes
Participant responsiveness	Intervention uptake depends on its acceptance by and acceptability to those receiving it. Low participant involvement or responsiveness may negatively impact intervention fidelity.
Recruitment^a^	Refers to procedures that were used to attract potential programme participants.
Context^a^	Refers to the surrounding social systems, such as structures and cultures of organizations and groups, and historical and concurrent activities and events

Adapted from Carrol et al. [24]

^a^These components were added by Hasson [36]

### Fidelity assessment in CRT reporting guidelines

The Consolidated Standards of Reporting Trials (CONSORT) group was created to provide guidance to improve the quality and transparency of reporting of RCTs [37]. The CONSORT Statement offers a checklist of essential items that should be included in reporting a RCT [37]. Due to the increasing use of CRT designs, the CONSORT group proposed a version of the CONSORT Statement for the reporting of cluster randomised trials in 2004 and updated these guidelines in 2012 [2, 38].

The CONSORT Statement recognises that the trial protocol for a given study may not have been followed fully for some trial participants for a wide variety of reasons, including failure to receive the entire intervention as planned [37]. Cases of protocol nonadherence may influence the interpretation and credibility of the results and thus the validity of the conclusions [19, 26, 39, 40]. To preserve the ability to make strong inferences about the intervention effect, CONSORT offers recommendations on how issues of nonadherence should be handled at the level of analysis. Specifically, it recommends that all participants randomised be retained in the analysis and analysed according to their original assigned groups, an approach known as “intention-to-treat” or “ITT” analysis. This approach ignores noncompliance, protocol deviations, and anything that occurs after randomisation. The rationale for the ITT approach is that random allocation procedures avoid bias when assigning interventions to trial participants and thus facilitate causal inference. Any exclusion of patients from the analysis risks compromising the randomisation and may lead to biased results. This ITT approach can be contrasted with a “per protocol” or “PP” analysis, which restricts the analysis to participants who fulfil the protocol in terms of eligibility, interventions, and outcome assessment [19, 26, 39, 40]. According to the CONSORT, although a PP analysis may be appropriate in some instances, due to the exclusion of participants, it should be considered as a non-randomised, observational comparison.

The CONSORT guidance on handling protocol nonadherence has been primarily developed in relation to individually randomised parallel group trials. However, reasons for protocol nonadherence in individually randomised RCTs may differ from those in CRTs. In a clinical trial setting, nonadherence depends largely on the actions of the trial participant (e.g. failure to adhere to therapy) and the treatment provider (e.g. failure to follow treatment protocol), which may in turn be related to issues such as treatment side effects and safety. In CRTs of public health interventions, protocol nonadherence may occur because complex interventions that include multiple components are delivered with poor fidelity. However, despite the scientific importance of protocol nonadherence, the current CONSORT guidelines for individually randomised parallel group trials [37] and CRTs [2, 38] offer no advice on the methods to assess its occurrence during the course of a trial.

### Rationale for undertaking this review

LMIC governments and other development partners have strengthened research and intervention efforts to support the UN Millennium Development Goals (MDGs) and Sustainable Development Goals (SDGs) agenda. As the global community intensifies the search for the best evidence on public health interventions to improve health and development outcomes in LMICs, CRTs have become an essential tool. Policymakers are interested in using the best available evidence to make decisions about the effectiveness of specific interventions in LMICs facing considerable budget constraints. Although CRTs have been widely implemented to evaluate public health interventions in both high-income countries and LMICs, country context, interventions, approaches, and outcomes may differ substantially between settings. We therefore limit our focus to LMICs.

As earlier methodologically-oriented systematic reviews have demonstrated, CRTs of complex public health interventions may be particularly at risk of experiencing protocol deviations and nonadherence, and these may compromise the validity of their findings [19, 20]. Although process evaluation techniques such as evaluation of implementation fidelity can help to assess the extent of these problems and mitigate their negative effects, current reporting guidelines for CRTs offer no specific guidance on the assessment of intervention fidelity within CRTs. Wide divergence in current practices is therefore likely. We will undertake a methodologically-oriented systematic review of current practices related to the assessment of intervention fidelity within CRTs of public health interventions in LMICs, with a view to informing the best practices for these CRTs. To our knowledge, no other systematic review has been conducted on this question.

### Objective

We will conduct a systematic review of the published scientific literature to study current practices concerning the assessment of intervention fidelity in CRTs of public health interventions in LMICs.

This review will address the following research questions:

1. Based on information from the trial registry (and the published study protocol, if applicable): What proportion of recent CRTs of public health interventions in LMICs planned to assess implementation fidelity (IF)?
2. Based on information from the published trial report (or a complementary document such as a published article, a grey literature report, or an online appendix reporting the assessment of IF), what proportion of recent CRTs of public health interventions in LMICs reported assessing IF?
3. For those studies that assessed IF, which fidelity components were examined, and which data collection methods were employed to assess each component?
4. Is there evidence of divergent practices between planned and reported studies, or of outcome reporting bias related to the assessment of IF?
  - Based on comparison of the results of questions 1 and 2, what is the overall agreement between planned and reported assessment of IF?
  - Are trial reports with negative findings for the ITT analysis more likely to report a PP analysis?
  - For the subset of studies that included both ITT and PP analyses, what is the overall agreement between ITT and PP analyses concerning the intervention’s effectiveness?
  - Does the magnitude of the intervention effect differ for PP as compared to ITT analyses?

To answer our research questions, we will first identify all CRTs from 2012 onwards of public health interventions conducted in LMICs with an available study protocol registered in a public trial registry. A given CRT will be included in the review if the protocol, the trial report, or both address IF. For each CRT included in the review, we will compare planned assessment methods for IF as described in the trial registry (and published study protocol, if applicable) with published methods and results from the main trial report (and related documents, if relevant). We will use a variety of measures to summarise the results.

## Methods

We describe the study methods in seven steps adapted from the 2015 Preferred Reporting Items for Systematic Reviews and Meta-Analyses (PRISMA)-P reporting guidelines for systematic reviews and meta-analysis protocols [41]. The PRISMA-P checklist is provided as an additional file (see Additional file 1). As this review focuses on methodological issues rather than on health-related outcomes, it was not eligible for inclusion in the PROSPERO registry [42]. In the event of protocol amendments, we will provide the date of each amendment, a description of the change, and the rationale for the change.

### Eligibility criteria

Studies will be selected from the peer-reviewed scientific literature according to the following study and report characteristics.

#### Study characteristics

##### Study designs

We will include all CRTs. For the purposes of this review, a CRT is defined as a trial in which intact social units or clusters of individuals, rather than independent individuals, are randomly allocated to intervention groups [38]. CRTs may include trials employing parallel group, stepped wedge, or factorial designs; cluster randomised adaptive trials; and cluster randomised pragmatic trials, among others. CRTs with an adaptive design allow modifications based on data accumulated following trial start, while preserving the integrity of the trial [37]. Pragmatic trials are designed to evaluate the efficacy of an intervention in routine clinical practice in order to maximise applicability and generalizability of the results of the study [43, 44].

##### Participants

Study participants will be human adults or children living in LMICs. LMICs will be defined according to the 2016 World Bank country classifications [45].

##### Interventions

This review focuses on “public health interventions”. We employ the definition of “public health” proposed in the World Health Organization (WHO) health promotion glossary as “The science and art of promoting health, preventing disease, and prolonging life through the organized efforts of society” [46]. Adapting the definition proposed by Rychetnik and colleagues, we define a public health intervention as a disease prevention or health promotion intervention applied to many, most, or all members in a community, which aims to deliver a net benefit to the community or population, as well as benefits to individuals [47, 48]. Public health interventions are distinguished from clinical interventions aimed at preventing or treating diseases at the individual level [47, 48].

In order to operationalise this definition and guide selection of specific studies, we will use the “Intervention Wheel”, a graphic model of population-based public health practice illustrated with specific examples, developed by the Minnesota Department of Health [49]. The intervention wheel provides 17 public health interventions, selected to meet five criteria. To be considered as public health interventions, interventions should as follows: (i) focus on entire populations (or particular subgroups within a population), (ii) be grounded in an assessment of community health, (iii) consider the broad determinants of health, (iv) emphasise health promotion and prevention, and (v) intervene at multiple levels [49]. We used these five criteria to aid in decisions concerning study inclusion.

According to Rychetnik and colleagues, public health interventions are inherently “complex, programmatic, and context dependent” and these characteristics raise challenges for their evaluation [47]. The assessment of intervention fidelity may be especially important for public health interventions, and this consideration underlies our choice to focus on them in this review.

##### Comparators

Comparators will be defined as planned per the original CRT. Given the nature of public health interventions and the pragmatic orientation of CRTs in LMICs, we anticipate that a large proportion of studies included in the review will define the comparison group as receiving the “usual care”.

##### Outcomes

The focus of this methodologically-oriented review is on comparisons of planned and reported outcomes related to IF. For studies that assessed IF in either the trial protocol or the main trial report, we will include both the study protocol and the main trial report. Recognising that word limits for scientific journal articles are highly constrained and that the current CONSORT reporting guidelines for CRTs do not require description of elements related to IF, we also decided to include CRTs reporting the assessment of IF in a complementary document such as a published article, an online appendix to the main paper, or a grey literature report, in lieu of reporting the assessment of IF in the main trial report. These elements will be verified by checking the bibliography for the main trial report and additional sources.

For the purposes of study selection, we considered that studies evaluated IF if they either proposed methods to assess or reported results related to the evaluation of at least one of the four key fidelity components: (1) content, (2) coverage, (3) frequency, and (4) duration. For CRTs taking an adaptive approach, we will consider if these trials respect pre-established decision rules regarding changes to their design. In addition, we will include all CRTs that reported a per-protocol analysis.

#### Report characteristics

##### Setting

Eligible studies will be implemented in LMICs as classified by the World Bank [45].

##### Availability of the study protocol

To ensure availability of a study protocol, we will include CRTs reporting a registration number in the abstract for any trial registry meeting the WHO criteria [50]. The WHO trial registration data set (TRDS) is an internationally agreed-upon set of items that provide information on the design, conduct, and administration of clinical trials. The WHO International Clinical Trials Registry Platform (ICTRP) facilitates the publication of the TRDS on a publicly accessible website, through a network of partner registries that have agreed to adopt the TRDS as a common standard. The TRDS will be used in this review to evaluate planned assessment of intervention fidelity, either alone, or in conjunction with a published study protocol. TRDS field 20 “Key secondary outcomes” is particularly pertinent for this assessment.

##### Publication dates

We will include studies for which the main trial report was published on or after January 1, 2012. We chose this date because the last update of the CONSORT Statement to improve reporting of CRTs was published in 2012, and we wanted to analyse current practices. No restriction will be applied to the publication date for the protocol.

##### Language

We will include studies published in English, Spanish, or French, which are languages known by the research team.

##### Exclusion criteria

We will exclude studies that are (i) not cluster randomised trials, studies that (ii) do not plan or report the assessment of IF and (iii) are not public health interventions, (iv) conducted in a high-income country as defined by the World Bank 2016 country classification [45], (v) are published before 2012, (vi) do not have a publicly available protocol for comparison, or (vii) for which only the protocol but not the main trial report has been published. Manuscripts will be also excluded if they are (viii) pilot studies, (ix) secondary reports of a main study for which the relevant findings were published prior to 2012, (x) studies published in a language other than English, Spanish, or French, or (xi) studies from the grey literature.

### Information sources and search strategy

Literature search strategies were developed in collaboration with an academic librarian experienced in conducting systematic review searches. Search strategies use Medical Subject Headings (MESH) and text words related to cluster randomised trials, developing countries, and public health interventions. The electronic database search was developed first for MEDLINE (Ovid) (for the full search strategies, see Additional file 2) and then adapted for the following electronic databases: EMBASE (Ovid), CINAHL (Ovid), PubMed, and EMB Reviews (Ovid). Search terms are a combination of “cluster-randomized”, “cluster analysis”, “health program”, “public health service”, “health education”, “public health”, “health promotion”, “health behavior”, “health knowledge/attitudes practice”, “Preventive health services”, “health care system”, “health education”, and “developing countries”. The search strategy will span the time period from January 2012 to May 2016 and will be updated towards the end of the review. Searches will be filtered to articles concerning humans and written in English, French, or Spanish. To augment this list, we will add relevant studies suggested by members of the systematic review team. Identified records will be uploaded into the EndNote reference management software (version X7.5.3, Thompson Reuters, 2016), and duplicates will be eliminated.

### Study screening and selection

Study screening and selection will be done manually within the EndNote based on the inclusion and exclusion criteria for this systematic review. To ensure the availability of study protocols, we will limit the search to CRTs that have the word stem “regist*” in the abstract and use these results to begin the process of screening and selection. We validated this procedure by examining a subset of excluded articles. Screening and selection will be done in two stages by two independent reviewers (MCP and NM). In the first stage, reviewers will independently screen the titles and abstracts of each identified reference against the inclusion criteria to eliminate irrelevant publications. In the second stage, we will screen the full text of all studies that appear to meet the inclusion criteria or for which there is uncertainty as to eligibility. For each study, we will identify additional articles of potential relevance, such as a published protocol or a process evaluation, by reviewing references from the main trial report, consulting the trial registry record, and searching the PubMed database for new publications by the lead trial author. To aid in article screening and selection, the team will develop and test a screening sheet for full-text review. Any disagreement between reviewers will be resolved through discussion and, as necessary, through arbitration by a third author (MJ). The process of study selection will be documented in a flow diagram describing studies identified and excluded at each stage. We will also provide a summary table describing studies excluded at the stage of full-text review, along with reasons for their exclusion.

### Outcomes and prioritisation

The search and selection process for this review is designed to identify two quantities required for calculation of outcomes based on proportions: (1) numerator: These are studies that meet all the inclusion and exclusion criteria. As for all systematic reviews, these studies are our principal focus and will be included in the review and given detailed analysis. (2) Denominator: This is the total *N* for the study, which we defined as all studies that satisfy all the inclusion and exclusion criteria, with the exception of the outcome criterion (planned or reported IF assessment). It is essentially the universe of cluster randomised trials of public health interventions in LMICs. Both quantities will be clearly indicated in the study flow diagram.

### Primary outcome

The primary outcome for this study will be the proportion of overall agreement between the protocol and trial report concerning occurrence of IF assessment. This corresponds to research question 4a.

Data will be summarised in a two-by-two table comparing the assessment of intervention fidelity in the trial report to that in the protocol. *N* represents the set of recent CRTs of public health interventions in LMICs that have registered the study protocol in a publicly availably trial registry. For each CRT in *N*, we will determine whether IF was assessed in the registered (or published) protocol or in the trial report (or associated documents). Studies judged to have assessed IF will be coded as “1”; others will be coded as “0”. Judgements will represent reviewer consensus (MCP and NM, with appeal to MJ in case of divergences). The proportion of overall agreement is defined as the proportion of eligible CRTs for which judgements concerning the occurrence of implementation fidelity assessment agree in the protocol and in the trial report (i.e. both positive or both negative). It will be computed as (*a* + *d*)/*N*.

**Table:** 

		Protocol		
		+	−	
Trial report	+	*a*	*b*	*a* + *b*
	−	*c*	*d*	*c* + *d*
		*a* + *c*	*b* + *d*	*N*

### Secondary outcomes

To address research questions 1, 2, and 3, we will also calculate the following:

- The frequency and proportion of trial *protocols* reporting the assessment of intervention fidelity, out of *N*
- The frequency and proportion of trial *reports* reporting the assessment of intervention fidelity, out of *N*
- The proportion of positive agreement among those that agree, computed as *a*/(*a* + *d*)
- The frequency counts and percentages summarising fidelity components examined and data collection methods proposed or employed

To address research question 4b, for all studies included in the trial, we will also record the authors’ judgments as to whether the intervention was effective. Studies that concluded that the intervention is more effective than the control will be coded as “1”; studies that were unable to reject the null hypothesis that there are no significant differences between groups will be coded as “0”. We will calculate as follows:

- The conditional probability that a PP analysis is performed given that the ITT analysis shows no difference between groups.
- The conditional probability that a PP analysis is performed given that the ITT analysis shows a positive intervention effect.

These measures will be calculated using a standard formula for conditional probabilities:

$$ P\left(B\Big|A\right)=\frac{P\left(A\ \mathrm{and}\ B\right)}{P(A)} $$

To address research questions 4c and 4d, we will examine the subset of trial reports containing both ITT and PP analyses. For studies comparing several interventions (e.g. factorial design), data on each intervention will be extracted separately.

To address research question 4c, we will study the proportion of the overall agreement between the ITT and PP analyses concerning intervention effectiveness.

Data will be summarised in a two-by-two table comparing the assessment of intervention effectiveness in the ITT analysis to that in the PP (intervention fidelity) analysis. *T* is the total number of included CRTs reporting both an ITT and PP analysis. Studies that concluded in favour of the intervention group will be coded as “1”; those that are unable to reject the null hypothesis that there is no significant difference between groups will be coded as “0”. Judgements will represent reviewer consensus (MCP and NM, with appeal to MJ in case of divergences). The proportion of overall agreement is defined as the proportion trial reports for which judgements concerning intervention effectiveness agree in ITT and PP analyses (i.e. both positive (favour the intervention group) or both negative (unable to reject the null hypothesis of no difference between groups)). It will be computed as (*w* + *z*)/*T*.

**Table:** 

		ITT analysis		
		+	−	
PP analysis	+	*w*	*x*	*w + x*
	−	*y*	*z*	*y + z*
		*w + y*	*x + z*	*T*

We will also calculate

- The frequency and proportion of ITT analyses that conclude in favour of the intervention, out of *T*
- The frequency and proportion of PP analyses that conclude in favour of the intervention, out of *T*

To address research question 4d, we will compare intervention effect sizes reported for ITT and PP analyses. Comparisons will be summarised as the percentage change in effect size, computed as the effect size for the PP analysis/effect size for the ITT analysis *100.

### Risk of bias in individual studies

To assess possible risk of bias for included studies, we will use the Cochrane Collaboration tool to assess the risk of bias in randomised trials [51] based on the following factors: random sequence generation, allocation concealment, blinding of participants and personnel, blinding of outcome assessment, incomplete outcome data, selective reporting, and other sources of bias. Because the Cochrane Collaboration tool was developed for individually randomised studies whereas our study focuses on CRTs, we will also include several additional criteria specifically relevant to assessing risk of bias in CRTs, recommended by the Cochrane Collaboration [51] and other key sources [51–53]. These additional criteria will consider issues related to the following: recruitment bias (potential for participant self-selection to occur if individuals are recruited to the trial after the clusters have been randomised); baseline imbalances (because CRTs generally randomise a limited number of clusters, chance imbalances may affect comparability of intervention and control groups); loss of clusters (complete clusters may sometimes be lost from a trial and thus be omitted from the analysis; these missing data may lead to biased outcome assessments); and unit of analysis (failure to properly account for clustering in the analysis) [51]. For each domain or criterion of interest, we will assess each criterion as low risk, high risk, or uncertain risk and provide a sample text that illustrates the reasons for this judgement. This evaluation will be done independently by two reviewers (MCP and NM). Disagreements between reviewers will be resolved by consensus or, if consensus cannot be achieved, by consulting a third reviewer (MJ). Judgements related to risk of bias will be summarised graphically using RevMan 5.1 [51]. Risk of bias assessments will be used to create categories of high-, uncertain-, and low-risk studies to be used in subgroup analyses.

Systematic reviews of health outcomes often assess the quality of a body of evidence using standardised tools such as the GRADE system [54]. However, as this review focuses on methodological issues rather than on health-related outcomes, we will not use this tool.

### Data extraction and data items

Two review authors will extract data independently (MCP and NM). From each study protocol and trial report, reviewers will extract data on (i) the study characteristics (study location, aims, intervention); (ii) all applicable descriptors of the CRT trial design (for example, parallel group, stepped wedge, factorial, adaptive, pragmatic); (iii) concepts related to the assessment of IF (assessment of fidelity reported in protocol and/or main study, fidelity components and moderating factors evaluated, data collection methods, and any dimension used by the authors to evaluate intervention fidelity distinct from those proposed by Caroll and Hasson [24, 32]); (iv) whether events taking place in the control group were monitored, as these can influence the effectiveness of the intervention [27, 55, 56]; and (v) information for assessing the risk of bias of included studies. We will also extract (vi) statistical results concerning the intervention effectiveness and the authors’ qualitative conclusions regarding the intervention effect for the primary (generally, ITT) analysis and one or more subgroup analyses relevant for intervention fidelity (generally, the PP analysis). If studies investigate more than one intervention, we will extract data relevant for each comparison. To reduce bias and errors in data extraction, reviewers will use a pre-defined template pilot tested on a subset of studies and a guide for data extraction. To ensure consistency, reviewers will receive training prior to commencing extraction for the review and undertake calibration exercises. Reviewers will resolve disagreements by discussion and by appeal to a third author (MJ) where necessary. All data extraction tools will be available as online supplementary documents.

### Data synthesis

Results will be presented in accordance with the PRISMA Statement [41]. A narrative synthesis will be provided, with information presented in tables to summarise key data. The narrative synthesis will explore relationships and findings within and between the included studies. It will highlight the four key dimensions of intervention fidelity identified from the literature (content, coverage, frequency, and duration), moderating factors for intervention fidelity (participant responsiveness, comprehensiveness of policy, strategies to facilitate implementation, quality of delivery, recruitment, and context), any new dimensions explored, and data collection method used to evaluate each key dimension.

We will present quantitative data for all primary and secondary outcomes proposed. Where appropriate, data will be presented in tabular form.

We will investigate the possible sources of heterogeneity by performing subgroup analysis. Specifically, we will recompute the main quantitative outcomes for subgroups of studies with high, uncertain, and low risk of bias to better understand potential sources of variation in results. If the data permit, we will conduct a sensitivity analysis to explore whether studies at lower risk of bias undertake more comprehensive assessment of intervention fidelity. Because of the study question and the nature of the outcomes assessed, we do not intend to perform meta-analyses.

### Planned assessment of meta-biases

We recognize that data may be biased due to non-study-related processes and plan to assess specific meta-biases. This study compares results for protocols and published trial reports, and is thus designed to address potential reporting bias and to investigate potential outcome bias. As our review focuses on methodological issues rather than on outcome assessment, we will not assess potential publication bias.

## Discussion

Development initiatives require high-quality evaluations to determine whether the programmes work or not and to know how to improve them [57, 58]. According to Rychetnik et al. [48], evaluation of public health interventions requires detailed information about the “design and implementation of an intervention; contextual circumstances in which the intervention was implemented; and how the intervention was received”.

We will conduct a methodological systematic review to evaluate the current practices for evaluating implementation fidelity in CRTs of public health interventions carried out in LMICs. Fidelity assessment may be a key tool for making studies more reliable, internally valid, and externally generalizable [59]. In the absence of fidelity assessment, it may be difficult to determine if CRT results are due to the intervention design, to its implementation, or to unknown or external factors that may influence results. The rejection of effective interventions or acceptance of ineffective interventions incurs incalculable costs, due to the use of financial and scientific resources, and the inability of the authors to extrapolate the results [26]. Improved assessment and reporting of intervention fidelity may be important for researchers, for those who finance health interventions, and for decision-makers who seek the best evidence on public health interventions to promote health, prevent disease, and reduce health inequalities.

## Additional files

Additional file 1: Comparison of registered and published assessment of intervention fidelity in cluster randomised trials of public health interventions in developing countries: systematic review protocol. Preferred Reporting Items for Systematic review and Meta-Analysis Protocols (PRISMA-P) 2015 checklist: recommended items to address in a systematic review protocol*. (DOCX 134 kb)
Additional file 2: The search strategy for MEDLINE. (DOCX 102 kb)

## Abbreviation

CONSORT: Consolidated Standards of Reporting Trials
CRTs: Cluster randomization trials
ICTRP: International Clinical Trials Registry Platform
IF: Intervention fidelity
ITT: Intention-to-treat
LMICs: Low- and middle-income countries
MESH: Medical Subject Headings
PP: Per protocol
PRISMA: Preferred Reporting Items for Systematic Reviews and Meta-Analyses
RCTs: Randomised controlled trials
TRDS: Trial registration data set
WHO: World Health Organization
