# Supplementary material for: Comparison of registered and published intervention fidelity assessment in cluster randomised trials of public health interventions in low- and middle-income countries: systematic review protocol
Source: Syst Rev. 2016 Oct 19;5:177. doi: 10.1186/s13643-016-0351-0 (PMC5069975; doi:10.1186/s13643-016-0351-0)
Supplement: Additional file 2: — The search strategy for MEDLINE. (DOCX 102 kb) [file 13643_2016_351_MOESM2_ESM.docx]

**Additional file 2**

**The search strategy for MEDLINE**

Database: Epub Ahead of Print, In-Process & Other Non-Indexed Citations, Ovid MEDLINE(R) Daily and Ovid MEDLINE(R) 1946 to Present Interface: OvidSP

Research date: May 13^th^, 2016

Filters 2012 onwards, English, French, Spanish

| 1 | Cluster Analysis/ | 49336 |
| --- | --- | --- |
| 2 | Cluster Analys*.tw,kw,sh. | 59132 |
| 3 | (communit* adj2 random* adj2 trial*).tw,kw,sh. | 630 |
| 4 | (group adj2 random* adj2 trial*).tw,kw,sh. | 2007 |
| 5 | (cluster adj2 random*).tw,kw,sh. | 7944 |
| 6 | cluster-random*.tw,kw,sh. | 6686 |
| 7 | **or/1-6** | **67832** |
| 8 | Afghanistan/ or Libya/ or Albania/ or Macedonia Republic/ or Algeria/ or Madagascar/ or American Samoa/ or Malawi/ or Angola/ or Malaysia/ or Argentina/ or Indian Ocean Islands/ or Armenia/ or Mali/ or Azerbaijan/ or Micronesia/ or Bangladesh/ or Mauritania/ or Republic of Belarus/ or Mauritius/ or Belize/ or Mexico/ or Benin/ or Bhutan/ or Moldova/ or Bolivia/ or Mongolia/ or Bosnia-Herzegovina/ or Montenegro/ or Botswana/ or Morocco/ or Brazil/ or Mozambique/ or Bulgaria/ or Myanmar/ or Burkina Faso/ or Namibia/ or Burundi/ or Nepal/ or Nicaragua/ or Cambodia/ or Niger/ or Cameroon/ or Nigeria/ or Central African Republic/ or Pakistan/ or Chad/ or Palau/ or China/ or Panama/ or Colombia/ or Papua New Guinea/ or Comoros/ or Paraguay/ or Democratic Republic of the Congo/ or Congo/ or Peru/ or Philippines/ or Costa Rica/ or Romania/ or Cote d'Ivoire/ or Rwanda/ or Cuba/ or Samoa/ or Djibouti/ or Atlantic Islands/ or Dominica/ or Senegal/ or Dominican Republic/ or Serbia/ or Ecuador/ or Seychelles/ or Egypt/ or Sierra Leone/ or El Salvador/ or Melanesia/ or Eritrea/ or Somalia/ or Ethiopia/ or South Africa/ or Fiji/ or Sudan/ or Gabon/ or Sri Lanka/ or Gambia/ or Saint Lucia/ or Georgia/ or "Saint Vincent and the Grenadines"/ or Ghana/ or Grenada/ or Suriname/ or Guatemala/ or Swaziland/ or Guinea/ or Syria/ or Guinea-Bissau/ or Tajikistan/ or Guyana/ or Tanzania/ or Haiti/ or Thailand/ or Honduras/ or East Timor/ or Hungary/ or Togo/ or India/ or Tonga/ or Indonesia/ or Tunisia/ or Iran/ or Turkey/ or Iraq/ or Turkmenistan/ or Jamaica/ or Micronesia/ or Jordan/ or Uganda/ or Kazakhstan/ or Ukraine/ or Kenya/ or Uzbekistan/ or Vanuatu/ or Korea/ or Venezuela/ or Kosovo/ or Vietnam/ or Kyrgyzstan/ or Middle East/ or Yemen/ or Lebanon/ or Lesotho/ or Zambia/ or Zimbabwe/ or Liberia/ or Developing Countries/ or Poverty/ or Rural Population/ | 823062 |
| 9 | (Afghanistan or Albania or Algeria or American Samoa or Angola or Argentina or Armenia or Azerbaijan or Bangladesh or Belarus or Belize or Benin or Bhutan or Bolivia or Bosnia Herzegovina or Botswana or Brazil or Bulgaria or Burkina Faso or Burundi or Cabo Verde or Cambodia or Cameroon or Central African Republic or Chad or China or Colombia or Comoros or Congo or Costa Rica or Cote d'Ivoire or Cuba or Djibouti or Dominica or Dominican Republic or Ecuador or Egypt or El Salvador or Eritrea or Ethiopia or Fiji or Gabon or Gambia or Georgia or Ghana or Grenada or Guatemala or Guinea or Guinea-Bissau or Guyana or Haiti or Honduras or Hungary or India or Indonesia or Iran or Iraq or Jamaica or Jordan or Kazakhstan or Kenya or Kiribati or Korea or Kosovo or Kyrgyz or Lao PDR or Lebanon or Lesotho or Liberia or Libya or Macedonia or Madagascar or Malawi or Malaysia or Maldives or Mali or Marshall Islands or Mauritania or Mauritius or Mexico or Micronesia or Moldova or Mongolia or Montenegro or Morocco or Mozambique or Myanmar or Namibia or Nepal or Nicaragua or Niger or Nigeria or Pakistan or Palau or Panama or Papua New Guinea or Paraguay or Peru or Philippines or Romania or Rwanda or Samoa or Sao Tome or Senegal or Serbia or Seychelles or Sierra Leone or Solomon Islands or Somalia or South Africa or South Sudan or Sri Lanka or St Lucia or Sudan or Suriname or Swaziland or Syrian Arab Republic or Tajikistan or Tanzania or Thailand or Timor Leste or Togo or Tonga or Tunisia or Turkey or Turkmenistan or Tuvalu or Uganda or Ukraine or Uzbekistan or Vanuatu or Venezuela or Vietnam or Gaza or Yemen or Zambia or Zimbabwe or Developing Countr* or middle income countr* or low income countr*).tw,kw. | 792287 |
| 10 | **8 or 9** | **1172790** |
| 11 | ((public adj2 health) or (poverty adj2 economic*) or interven* or program* or approach*).tw,kw,sh. | 2459336 |
| 12 | Public Health/ or Community Health Services/ or Community Health Workers/ or Community Health Planning/ or Community Networks/ | 107616 |
| 13 | **11 or 12** | **2515327** |
| 14 | **7 and 10 and 13** | **4068** |
| 15 | **remove duplicates from 14** | **3964** |
| 16 | **limit 15 to yr="2012 -Current"** | **2069** |
| 17 | **limit 16 to (english or french or spanish)** | **2024** |
